# Medical and Physical Intelligence
*To prevent an accumulation of deferred Original Communications, and the appearance of neglect towards our Correspondents, we have been obliged to bring some of the shortest into this department.—Editors.


**Published:** 1825-02

**Authors:** 


### MEDICAL AND PHYSICAL INTELLIGENCE.*

### PATHOLOGY.

1. *Fatal Case of irritative Fever, produced by a Scratch on the Finger;* by JOHN ANDERSON, Esq. &c. &c.—MRS. C., aged forty, of a strong, healthy constitution, and mother of a large family, whilst in the daily practice of washing and dressing a suppurating wound in the neck of one of her children, complained of pain and tenderness in the last joint of the middle finger of her right hand; the knuckle of which was swelled and inflamed, and on the inner side there appeared the remains of a slight scratch or wound, of which she could give no account. She was desired to apply the liq. plumbi acet. dilut. to the part affected, and on my next visit the pain and inflammation had subsided. The following day she felt considerable pain above the clavicle on that side, extending to the ear, although there was no external appearance of inflammation in that part, nor any tenderness in the arm or finger first affected. As she had become rather feverish, she took some purgative medicine, and afterwards the mist. salin. cum vin. antim.

The next day, the pain had increased along the side of the head; and the same night she was seized with delirium, which, however, left her in the morning. The pain above the clavicle had now subsided, and she complained only of a slight head-ach and want of sleep; her pulse was about ninety, and her skin cool and moist. She passed the day very tranquilly, but again became delirious through the night; which continued at intervals during the following day, when she was seen by Dr. Babington, who, upon learning the history of the case, proposed that Mr. Travers should be consulted the next morning.

When we visited Mrs. C. the following day, we found her very delirious, with a rapid pulse and constant watchfulness. Mr. Travers, conceiving that the present symptoms might arise from irritation of matter under the sheath of the tendon, made a deep incision into the joint of the finger, from whence a very small quantity of matter was evacuated; but the symptoms were not relieved by the operation. She was bled, and took purgative medicines, which latter acted very freely; and at night a large dose of opium was administered, but without procuring her any sleep. Twenty-four leeches were applied to the hand and arm, which were constantly kept fomented and poulticed. On the next day she was again bled, and took the mist. camph. cum ext. hyoscyam. every four hours; but without experiencing any diminution of the delirium, and without producing sleep.

In this manner she continued for some days, when, finding her strength much reduced, she was ordered dec. cinch. cum acid. sulph. dilut. Her restlessness and incessant delirium continued to increase, until she died, which event took place about three weeks from her first complaining.

---

* To prevent an accumulation of deferred Original Communications, and the appearance of neglect towards our Correspondents, we have been obliged to bring some of the shortest into this department.—EDITORS.

Upon examining the hand and arm after death, the tendons in the palm of the hand were found in a sloughy state, and a collection of matter had formed under the ligament. annul. of the wrist; but the whole of the arm appeared free from disease.

In this case a very remarkable circumstance occurred: the servant, who fomented and poulticed Mrs. C.'s hand, in a few days after the finger was opened, was attacked with violent inflammation and swelling of the hand and fingers, afterwards extending to the arm; and several collections of matter were formed, which were opened. Her health suffered so severely from this attack, as to excite for some time considerable apprehensions for her life.

*Fleet-street; December 12th,* 1824.

2. ***Pustules found under the Tongue in Cases of Hydrophobia.***—Having seen the notice, in a late Number of your Journal, respecting the pustules found under the tongue of patients affected with hydrophobia, as mentioned by Marochetti, I have taken the liberty of sending you the following copy of a written paper, which came accidentally into my hands about six years ago:—

"The inhabitants of Gadior have made the discovery, that, near the ligament of the tongue of the man or animal bitten by a rabid animal, and becoming themselves rabid, pustules of a whitish hue make their appearance: they open spontaneously about the thirteenth day after the bite, and at this time it is that the first appearance of the symptoms of hydrophobia occur. The method of cure consists in opening these pustules, and making the patient spit out the ichor and fluid of which they are then formed, often washing the mouth with salt water. This operation should be performed the ninth day after the bite. The remedy is so effectual amongst this people, that this hitherto incurable disease has entirely lost its terrors."—*(From* Dr. Davis's *Travels in Africa.)*

Never having seen Dr. Davis's book, I do not know who he was, nor in what part of Africa he travelled.

I have merely sent this as another proof of the widely-diffused belief in these pustules.—(*Private Note, signed* T. K.)

*December* 16, 1824.

3. *Sugar in the Blood of Diabetic Patients.*—MM. Vauquelin and Legelas d'Etchepare relate, that, taking advantage of the admission of a woman, fifty years of age, labouring under diabetes, into the Hôtel Dieu, under the care of Dr. Asselin, they determined to put to the proof the assertion made in the Dictionnaire des Sciences Medicales, article *Diabetes,* relative to the presence of sugar in the blood of patients affected with this disease; an assertion which, although in conformity with the theoretical views of certain physiologists, is in direct contradiction to the results obtained by many skilful experimentalists, particularly MM. Dupuytren and Thenard. In consequence of inflammatory symptoms rendering two large bleedings necessary, the blood was analysed twice with the utmost care; but it was not possible to discover a single atom of sugar, although the urine which the patient passed, to the amount of nine or ten pints in the day, contained a seventh part of this substance. The saliva, also, examined at two different times, was entirely free from all trace of sugar.

The author of the article in the Dictionary having proposed urea as a remedy in this disease, the patient was put upon the use of it for several days; and the urine made during its exhibition was analysed, in the hopes of discovering some traces of it. But the search was in vain: the urine retained its morbid qualities, but was considerably augmented in quantity. It is scarcely necessary to observe, that this result does not prove that the sugar is formed by the kidneys. It may, in fact, exist in the blood, and yet may not be detectable by our methods of analysis, as was demonstrated in the case of urea, by the labours of MM. Prevost and Dumas, as well as our own.—(*Revue Medicale, Fran. et Etrang.* Decembre.)

4. *Mercury in the Urine.*—Dr. CANTON has made the following experiments:—Sixty pounds of the urine of syphilitic patients, under the use of mercurial frictions, became alkaline in a short space of time, giving an abundant precipitate, which was separated by filtration. The fluid, subjected to various trials, did not afford any indication of the presence of mercury. The precipitate, mixed with equal weights of charcoal and carbonate of potash, and a sufficient quantity of water to form a paste, was submitted gradually to a red heat in a glass retort, the neck of which was plunged into a receiver filled with water. At the termination of the experiment, a pulvurulent precipitate was observed, which, after having been dried, gave out globules of quicksilver by simple compression: the quantity appeared to amount to more than twenty grains. The neck of the retort also contained a considerable number of small globules.—(*Memoires de Turin.*)

5. *Chronic Affection of the Brain.*—M. CAYOL relates the case of a man, a joiner by trade, thirty-one years of age, whose lower jaw had been fractured by a gun-shot wound, eight years prior to his present attack. Since that period he was seized, from time to time, but at very uncertain intervals, with a sudden giddiness, which caused him to fall down, if there was nothing within his reach by which he could support himself: at the same time, his vision was disturbed, his face was red and swollen. For the last six months, however, the disease had changed its form: he experienced a dull and heavy deep-seated pain, which was suddenly felt in the sole of his foot, and mounted immediately, along the leg and thigh, into the right side. These attacks, which lasted five or six minutes, were always accompanied with pains in the head and giddiness. He had, besides, habitually after his meals, a feeling of heat at the epigastrium, with hot and acid eructations.

M. Cayol, considering this disease as an affection of the nerves, tending to epilepsy, and caused probably by some organic lesion of the brain, prescribed bleeding from the arm, leeches to the anus, sinapisms on the lower limbs, and the repeated use of calcined magnesia, in such doses as to act as a purgative. The symptoms of pyrosis gave way speedily; and, as he had no return of his usual pains for eight days, his appetite, &c. being good, he chose to return to his employment.—(*Revue Medicale, Franc. et Etrang.* Novembre.)

### THERAPEUTICS.

6. *Effects of the Hot Bath continued for too long a time.*—A woman, twenty-eight years of age, had suffered during six months from an attack of rheumatism in the wrists and ankles, which had been relieved by the application of leeches, poultices, and by warm baths and frictions. A quack recommended the patient to remain in a bath heated progressively to nearly the boiling point, for the space of twelve hours. She went into the bath at noon, where she remained for six hours, and then lost her recollection: an hour after, she was found senseless, her head resting against the board which covered the bath. She was taken out of the water. The face was enormously swollen and blackish, the eyelids tumified; there were frothing at the mouth, grinding of the teeth, convulsions of the limbs; laborious respiration; hard, frequent, and irregular pulse. A copious bleeding restored the faculties of the mind and the speech: she complained of pain in the epigastrium, which was relieved by the application of forty leeches. The next day, though better, there was pain round the umbilicus, and twenty more leeches were applied. A perfect cure was effected; but the whole of the cuticle peeled off at the end of six weeks.—(*Journal Universel,* Novembre.)

7. *Bark of the Pomegranate Root a Remedy for Tænia.*—M. Husson has communicated to the Royal Academy of Medicine another instance of the success of the above remedy. The patient had been troubled with symptoms of tænia for nearly ten years, and had tried many remedies in vain. The medicine was prepared by the patient, by putting two ounces of the bark of the root in a pint and a half of water, which was boiled down to a pint; and the dose was two ounces, taken every two hours. At the end of the day, the patient, having suffered violent cholicy pains, felt a kind of flat ribband escape from the anus, which, in his haste to get rid of, he broke: the portion broken off was part of a tænia, three ells in length. The patient is to have recourse to the same remedy, as soon as he has again passed some portions of the worm; which circumstance is indispensable in the administration of the medicine, for it has never been found to be productive of benefit until the patients have passed for some time portions of the tænia.—(*Revue Medicale, Fran. et Etrang.* Novembre.)

8. *Utility of fresh Vegetables in Fever, when placed near the Patient.*—We have received a letter from Mr. S. Shatte, of Stokenham, Devon, the purport of which is to call to our notice a practice formerly recommended by some of the ancient writers on medicine, and which, he says, he has found of great service in fevers of the typhoid kind. A short extract from our venerable correspondent's letter, will explain his meaning sufficiently.

"In 1821, when typhous fever was very prevalent in this neighbourhood, I made several trials of this remedy. G. F., in the village of Chillington, who was taken ill with that disease, having a wife and two children, with only two small rooms up stairs: I immediately had the rooms and the bed covered with wet bushes or boughs of ash, hazle, willow, or any green shrubs that could be procured. The old ones were carried out, and fresh ones brought in every morning; and I am of opinion that, when they are brought in with the dew upon them, they are more efficacious: at all events, they must be made very wet with cold water, and be in considerable quantities, so as to cover the whole room."

In one case, our correspondent adds that they appeared to revive a patient almost at the point of death.

Mr. S. adds several quotations from Fernelius, Nic. Fontano, &c. who advocate the same practice; and finally he objects, and we think with great reason, to the abstraction of large quantities of blood in pure fever likely to assume a typhoid character.

9. *Remarks on Nævi.*—In the Journal for October, a correspondent, signing himself NÆVUS, desired to be informed, through the medium of this publication, of a remedy for nævi materni, or mother's marks. I am not physiologist enough to determine whether the mother can really exert that power over the fœtus in utero, so as completely to alter the formation or structure of it. There is no law, that I am acquainted with, which can give to her that peculiar power. Having premised thus much, I now mention the plan of treatment to be pursued in curing these marks. I would recommend a neat incision being made of the part affected, and allowing granulations to spring up as quickly as possible; or, if the patient mentioned by Nævus has not yet been submitted to the vaccine disease, I would recommend the insertion of lymph under the part, or parts, so affected. Nævus may feel satisfied that, in the space of six weeks or two months, not a vestige of the complaint would remain.—(*Letter signed* A BOROUGH STUDENT.)

10. *Remarks on Snuff-taking, with reference to* Dr. KINGLAKE's *Paper in this Journal for December.*—In the fifth article of your Journal for December, I was gratified by the attack which Dr. Kinglake made on snuff-takers. His reasoning, as usual, was very good. No person of common sense would attempt to justify a practice, which is not only uncleanly, but frequently gives rise to dyspepsia.

I regret extremely that, in the midst of his triumph, he should do such mischief to his cause, by introducing the case of the most illustrious personage of the age.

Dr. K. says, "There is much reason for believing that the ever-memorable Napoleon Bonaparte derived the cause of his protracted suffering, and eventual death, from the large quantities of snuff which he lavishly, but unconsciously, carried into his stomach through the nostrils, by the habit of strong and unmeasured inspiration with which he used that destructive agent."

Now, I do not think that there is any reason for believing that the small quantity of snuff which finds its way into the centre of sympathy, (for I never found much in any stomach, though I have had an opportunity of seeing hundreds of dissections in France, where this nasal stimulant is taken in such large quantities,) is sufficient to produce those cancerous appearances, which were evident in the case alluded to.

As the ingenious Doctor, who rides his hobby so hard, will no doubt ask me the following question, I beg leave to anticipate it: "If tobacco did not occasion the disease in question, what was the true cause of it?"

I answer, that anxiety was the exciting cause, and that Napoleon died of what is generally called a broken heart,—but, more properly, a broken-up stomach. Every body knows that a few hours' grief will derange the digestive organs more than a month's indulgence in that disgusting habit which the Doctor so properly reprobates; and every surgeon must be aware that distress of mind, where there is a predisposition to scirrhus or cancer, excites these baneful diseases.

During my studies at the Borough Hospital, I observed that the first question that Sir A. Cooper put to those afflicted with the above diseases, was concerning their late state of mind; and I may say that, during three years, I always noted the answer to be this—" Previous to the appearance of this disease, I suffered considerable mental affliction."

Dr. K. forgot to tell us, also, that Bonaparte drank more coffee than any other man in Europe. I contend that the free use of this beverage was sufficient antidote for the tobacco: but I will not say that the immoderate use of it, when the mind was constantly in a state of great excitation, did not aggravate that disease which deprived the world of the greatest hero ever known.—(*Letter from* Mr. ECCUM, *Surgeon*, 116, *Blackfriars-road*.)

---

### SURGERY.

11. *Remarks on Lateral Curvature of the Spine.*—As public attention has been latterly directed to the disease termed lateral curvature of the spine, in consequence of the increase of that most distressing disorder, the darkness in which its causes and mode of treatment were enveloped till within the last few years, and even now,—the difference of opinions which still prevails,—shows that we are far from having a perfect knowledge of the disease; I have taken the liberty to submit to your perusal the following remarks, which, should you think worthy of insertion in your Journal, will afford much pleasure to the writer.

It is well known that there is a perpetual change, by regeneration and absorption, occurring in all parts of the body; and, as bone is supposed to be secreted by the arteries, and removed by the absorbents, so the healthy action of each class is necessary for its continuance in a sound state. If the earthy constituent be deposited in the bones in excess, fragility, more or less, is the consequence: on the other hand, as rickets depends on a deficiency of nutrition, from the arteries not secreting earthy matter equivalent to the action of the absorbents, so I should consider the affection of the spinal column, termed lateral curvature, which arises chiefly in sedentary females, at a period when the ossification of the bones is not completed, to be a state analogous to that of rickets, brought on by an imperfect secretion of the constituents of bone in adequate proportions, which is caused principally by a neglect of due exercise of the spine, and differing from rickets only in degree.

The first curve takes place near the pelvis, to the left side; most probably in consequence of the yielding of the spine, from its not being able to support the superincumbent weight of the body: but the second curve of the dorsal vertebræ to the right side, which is also accompanied with a degree of rotation, is not dependent upon the same cause which produced the first curve, but owing to the muscles of that side (chiefly a portion of the trapesius, the longissimus dorsi, spinalis dorsi, and rhomboideus,) endeavouring to regain their lost balance, from having been placed on the stretch by the primary curvature.

According to this view of the disease, while the general health is preserved by attention to the state of the alimentary canal and the exhibition of tonics, the chief points which require attention in the local treatment are the straightening of the spine, and the more perfect secretion of the healthy constituents of bone, by which, when straight, the spine may recover its usual firmness.

The means by which we may endeavour to attain the first object, are the use of the inclined plane, (which does not occasion so great a determination of blood to the head as the horizontal,) for the space of three or four hours; and the exercise of swinging suspended by the hands to a rope.

The means to be pursued for the attainment of the second object, are by exercise of various kinds,—as that of balancing a light weight on the head, or that of raising a weight suspended through a pulley fixed to the wall, by bowing the head, as recommended by Mr. SHAW; together with the use of friction and the like, which act by giving greater strength to the muscles, and inducing an increased vascular action, in consequence of which the more perfect secretion of the healthy elements of bone is accomplished.

It might appear presumptuous in me to have offered any opinion, after such able surgeons have considered the subject, as I am not able to speak from experience; but, as the opinions of authors vary so widely, and I am not aware of the disease having been before viewed in this light, I submit the foregoing remarks to the notice of the profession.—(*Letter signed* A MEDICAL STUDENT.)

---

### CHEMISTRY.

12. ***On the Preparation of Morphia.***—The following process, by M. HOTTOT, appears, from the description, far to surpass any other that has yet been recommended, both for the readiness with which pure morphia is obtained, and the quantity of the product. Opium is to be dissolved in so much water, as to yield a solution of a specific gravity not higher than 1012; a small quantity of ammonia is then to be added, just sufficient to precipitate the colouring matter of the solution. In consequence of the diluted state of the liquor, this readily falls to the bottom; the clear solution is then removed, and more ammonia added to it to precipitate the morphia. The alkali separates, and falls, upon standing, as a crystalline sediment, containing very little colouring matter: this, washed with cold water, and afterwards treated with alcohol of sp. gr. 847 or 837, and a little animal charcoal, according to its quantity, gave, by the first operation, a morphia so pure that it required no further solution in alcohol or in sulphuric acid. By this process a considerable quantity of morphia may be obtained in twenty-four hours, with very little expence of alcohol: and the only point necessary to be attended to, is to separate carefully the fatty matter which falls in the first instance, on adding a small quantity of ammonia, so that it may not be re-dissolved by the addition of the further quantity of alkali necessary to precipitate the morphia.

It was found that the product from the magnesian process was rarely so white and pure as that furnished by the above method; and, in comparative experiments, the ammonia process yielded the largest result. The quantities of substances used are given as follows:—Macerate a kilogramme (35 oz. 5 dr.) of opium in sufficient cold water to exhaust the residuum; evaporate the liquors, until of specific gravity 1012, or nearly so; when half cooled, add to the solution so much ammonia as will make it neutral, or very slightly alkaline, about eight grammes (123.5 gr.); allow the precipitated matter to separate; decant, and again add ammonia in sufficient quantity, about sixty-four grammes (988.5 gr.); leave it for about twelve hours to deposit; throw the precipitate on a filter, wash it with cold water, and then treat it with three kilogrammes (6 lb. 10 oz.) of alcohol of specific gravity .847, and sixty-four grammes (988.5 gr.) of animal charcoal; heat it in a sand bath, and, when the alcohol boils, filter it: as it cools, the morphium will crystallise, amounting in weight to from 350 to 472 grains. The alcohol, rectified, will serve for further operations.—(*Journal de Pharmacie*, x. 475.)

13. *Active Principle of Belladonna.*—M. Runge has ascertained that the narcotic principle of belladonna is destroyed, or so changed by alkaline solutions, as to lose its distinguishing property of causing dilatation of the pupil. This takes place when the solutions are weak, or even with lime water; so that this principle cannot be obtained by the usual process, alkaline substances being used. Magnesia, however, was found not to exert any action of this kind, and may therefore be resorted to in the process; but it is very advantageous to use it as a hydrate, and not calcined. It should be thrown down from sulphate of magnesia by potash, not in sufficient quantity to decompose the whole of the salt; the mixture added to the aqueous infusion of belladonna, and the whole evaporated by a brisk fire to dryness: the residue, which is readily dried and pulverised, is to be treated with highly rectified boiling alcohol. The clear yellow solution is to be evaporated spontaneously, and a crystalline mass is obtained, which slightly blues reddened litmus paper, dissolves in water, and produces extreme dilatation of the pupil. The salts formed by it with sulphuric, muriatic, and nitric acids, also produce the same effect on the eye.—(*Ann. de Chim.* xxvii. 32.)

14. *On the active Principle of Colocynth (Colocyntine).*—The following account is extracted from a paper by M. Vauquelin:—

Colocynth, treated with alcohol, yields the bitter substance much purer than when water is used. The alcoholic solution, evaporated, yields a very brittle substance, of a gold-yellow colour, which, when put into cold water, produces a solution, whilst white opaque filaments remain, which ultimately form a soft, semi-transparent, yellow mass, resembling some resins. If the aqueous solution be heated, it becomes turbid, and there form, both on its surface and at its bottom, yellow drops, resembling a fused resin; these, when cold, become hard and brittle. The solution, re-heated, is again rendered turbid; and this effect is produced until the whole is evaporated.

It is found, on examination, that the substance remaining undissolved by the water at first, is of the same nature as that dissolved, and may itself be dissolved in abundance of water. It appears that the first portion of water dissolves more than the second or third, from the presence probably of some acid taken up by it. A yellowish-brown extractive matter also appears to be present; for the first solution is much more coloured than the second, and the product of the second solution evaporated appears to be much purer than that of the first. If, by successive evaporations, the resin-like substance be separated, the last, or mother liquor, contains a substance considerably soluble in water, and but slightly troubled by infusion of galls: the turbidness is occasioned probably by the presence of the bitter substance, a portion, as is evident to the taste, remaining.

The bitter resin-like substance is slightly soluble in water; considerably so in alkaline solutions; abundantly precipitated by infusion of galls, but not at all by acetate of lead. When heated, it yields a white vapour, of which the taste is not bitter, and leaves a very bulky charcoal. Nitric acid dissolves it, and the acid is decomposed, acting upon the substance, although with difficulty. If the solution be diluted, part of the substance precipitates in very bitter white flocculi.

This substance, containing the bitterness of the colocynth, appears to M. Vauquelin to be a particular principle. It is very soluble in alcohol, less so by far in water, but giving a solution of extreme bitterness, and frothing on agitation. He proposes for it the name of Colocyntine. —(*Jour. de Phar.* 1824, 416.)

---

### MIDWIFERY.

15. *Remarks on some Points connected with Midwifery.*—In looking over JOHNSON's Quarterly Journal for June 1824, a case is recorded of *Sloughing of the Bladder after Parturition,* and said to have been successfully treated.

It appears to me that the title of this paper should rather have been Laceration of the Bladder and Urethra, by an indiscreet use of the forceps.

In almost all cases of sloughing of the bladder after severe labour, the patient has the power of retaining urine for several days after delivery; whereas, this patient complained of an excessive discharge from the vagina on the day after delivery. It is evident, therefore, in this case, that the bladder had not time to inflame and to slough.

In cases where it is impossible to introduce a catheter, how can any rational practitioner expect to be able successfully to introduce the forceps? Mr. Guthrie in this case has forgot to inform us of the fate of the child.

It is some consolation, however, to practitioners in midwifery, to know that lacerations of the bladder may be cured by the means used in this case.

In the same Journal, the question respecting *pressure on the perineum* has very much divided the opinions of practitioners.

Some of the worst cases of laceration in perineo have occurred to patients in hospital, who have been delivered before any assistance could be procured. It is clear, therefore, that mischief may be done, which cannot be attributed to the mismanagement of the attendant.

It has been long my opinion that the principal object of a prudent practitioner ought to be to press the head of the fœtus, in its passage during a pain, against the arch of the pubes, and thus to diminish the pressure on the perineum. This mode of proceeding I consider much more eligible than pressure made on the perineum itself.

There is also a case of *Sudden Parturition* recorded in the same Journal, which is so contrary to daily experience, that I know not what to think of it.

In all twin cases which I have met, there has been some interval between the birth of the first and second child. The existence of a second child is generally ascertained by the largeness of the abdomen, and a difficulty in getting away the after-birth of the first.

The fact of twin children being immediately dropped into a night-chair, more especially in a case of first pregnancy, creates matter of very serious doubt. Although I would not venture to say it is impossible, I do not hesitate to say that it is most highly improbable.—(*Letter from* Dr. COLLINS, *of Dublin.*)

---

### MISCELLANEOUS.

16. *Notice respecting the Case of* ANN COULTHURST, *lately inserted in this Journal.*—We have received a communication from Mr. LIDDELL, dated from Preston, in which he complains of a misrepresentation of the case of Ann Coulthurst, as recorded in this Journal some few months ago; and he begs us to insert an explanation of the circumstances of that case. Our correspondent informs us, that the girl was thrown from one of those circumgyrating machines usually brought to fairs and other festivals, and suffered a severe compound fracture of the leg, with dislocation of the ankle;—that, when carried to her residence, the medical gentleman who usually attended the family was sent for; Mr. Lodge also was present, with several other professional men. A discussion took place as to the propriety of attempting reduction, or performing amputation; but the former plan was, by the persuasion of Mr. Liddell, acceded to. That gentleman reduced the bones with great difficulty; ordered the application of strong antiseptic cataplasms, with the administration of opiates at night. The future care of the patient devolved upon the medical gentleman who always attended the family. Mr. L. shortly after left Preston.

17.—*Literary Notice.*—A complete edition of the works of the late Dr. BAILLIE, with an account of his Life, collected from the most authentic sources, will speedily be published by Mr. WARDROP.

---

### OBITUARY.

### METEOROLOGICAL JOURNAL,

*From December 20, 1824, to January 19, 1825.*

By Messrs. WILLIAM HARRIS and Co. 50, Holborn, London.

| | MOON. | Rain gauge | THERM. | | | BAROM. | | DE LUC'S HYG. | | WIND. | | ATMOSPHERIC VARIATIONS. | | |
|---|---|---|---|---|---|---|---|---|---|---|---|---|---|---|
| | | | 9 A.M. | MAX. | MIN. | 9 A.M. | 10P.M. | 9 A.M. | 10P.M. | 9 A.M. | 10P.M. | 9 A.M. | 2 P.M. | 10P.M. |
| Dec | | | | | | | | | | | | | | |
| 20 | ● | | 52 | 53 | 36 | 29.35 | 29.55 | 94 | 90 | W | WSW | Rain | Rain | Fine |
| 21 | | .08 | 46 | 52 | 49 | 29.38 | 29.22 | 84 | 95 | WSW | WSW | Sho'ry | Sho'ry | Sho'ry |
| 22 | | | 51 | 52 | 34 | 28.94 | 28.99 | 89 | 82 | WSW | NNW | Rain | Rain | Cloud. |
| 23 | | .32 | 35 | 48 | 36 | 29.65 | 29.88 | 79 | 76 | NW | W | Fine | Fine | Fine |
| 24 | | .20 | 38 | 40 | 48 | 29.40 | 29.70 | 80 | 86 | W | W | Rain | Cloud. | |
| 25 | | | 53 | 53 | 43 | 29.40 | 29.66 | 97 | 88 | W | W | | | |
| 26 | | | 45 | 49 | 40 | 29.82 | 30.06 | 74 | 80 | W | WSW | Fine | Fine | Cloud. |
| 27 | | | 52 | 53 | 49 | 29.84 | 29.73 | 91 | 93 | WSW | SW | Cloud. | | Sleet |
| 28 | ☽ | | 51 | 52 | 37 | 29.68 | 29.76 | 92 | 93 | SW | NNE | | Sho'ry | Rain |
| 29 | | | 38 | 40 | 45 | 29.98 | 30.13 | 91 | 90 | NNE | SSW | Fine | Fine | Foggy |
| 30 | | .58 | 50 | 52 | 47 | 30.04 | 30.20 | 96 | 94 | SW | WSW | Rain | | Fine |
| 31 | | | 49 | 55 | 50 | 30.07 | 30.05 | 87 | 84 | WSW | SW | Cloud. | Sleet | Cloud. |
| Jan. | | | | | | | | | | | | | | |
| 1 | | | 53 | 54 | 38 | 30.03 | 29.92 | 85 | 88 | W | SW | Fair | Fine | Fine |
| 2 | | | 44 | 44 | 45 | 29.86 | 30.00 | 84 | 79 | W | SWva. | Fine | Sho'ry | Fine |
| 3 | | | 48 | 48 | 49 | 30.22 | 30.03 | 84 | 90 | WSW | WSW | Fine | Fine | Sho'ry |
| 4 | ○ | | 52 | 47 | 35 | 29.81 | 30.09 | 94 | 87 | W | NE | Cloud. | Sho'ry | Cloud. |
| 5 | | .16 | 37 | 38 | 33 | 30.42 | 30.48 | 80 | 81 | NE | N | Fair | Cloud. | Fine |
| 6 | | | 34 | 41 | 40 | 30.54 | 30.47 | 86 | 90 | WNW | SW | T. Fog | Fine | |
| 7 | | | 43 | 44 | 38 | 30.40 | 30.34 | 92 | 92 | W | NW | Cloud. | Rain | |
| 8 | | .11 | 39 | 39 | 35 | 30.57 | 30.64 | 88 | 89 | N | NNW | Fine | | |
| 9 | | | 37 | 42 | 40 | 30.72 | 30.72 | 90 | 92 | NW | NNW | Fog | Cloud. | |
| 10 | | | 41 | 43 | 36 | 30.70 | 30.67 | 88 | 86 | N | NNW | Cloud. | Fine | |
| 11 | ☾ | | 38 | 42 | 33 | 30.60 | 30.56 | 86 | 88 | NW | NNW | Fine | Cloud. | |
| 12 | | | 35 | 42 | 36 | 30.55 | 30.50 | 94 | 84 | W | NW | Foggy | Fine | |
| 13 | | | 37 | 40 | 38 | 30.43 | 30.35 | 85 | 85 | NNW | WSW | Cloud. | Cloud. | Cloud. |
| 14 | | | 41 | 43 | 42 | 30.23 | 30.23 | 89 | 84 | W | SW | | | |
| 15 | | | 43 | 44 | 38 | 30.12 | 30.01 | 85 | 81 | WSW | S | | Fine | Fine |
| 16 | | | 45 | 46 | 37 | 29.76 | 29.47 | 92 | 95 | WSW | S | Rain | Rain | Rain |
| 17 | | .15 | 38 | 45 | 40 | 29.76 | 29.89 | 90 | 90 | W | SW | Foggy | Fine | Fine |
| 18 | | | 47 | 48 | 34 | 29.37 | 29.30 | 93 | 85 | SSW | WSW | Rain | Rain | |
| 19 | ● | .12 | 35 | 38 | 37 | 29.33 | 29.40 | 90 | 82 | W | W | Fine | Cloud. | |

The quantity of rain fallen in the month of December, was 2 inches and 71.100ths.

